# Development of human innate immune responses in a humanized mouse model expressing four human myelopoiesis transgenes

**DOI:** 10.3389/fimmu.2024.1419117

**Published:** 2024-09-27

**Authors:** Hannah Stocks, Elisabeth De Leeuw, Bart N. Lambrecht, Linos Vandekerckhove, Geert van Loo, Andy Wullaert

**Affiliations:** ^1^ Department of Internal Medicine and Pediatrics, Ghent University, Ghent, Belgium; ^2^ VIB-UGent Center for Inflammation Research, VIB, Ghent, Belgium; ^3^ HIV Cure and Research Center (HCRC), Ghent, Belgium; ^4^ Department of Biomedical Molecular Biology, Ghent University, Ghent, Belgium; ^5^ Cell Death Signaling Lab, Department of Biomedical Sciences, University of Antwerp, Antwerp, Belgium

**Keywords:** humanized mice, innate immunity, myeloid cells, cytokines, inflammasome

## Abstract

**Background:**

Dysregulated innate immune responses underlie multiple inflammatory diseases, but clinical translation of preclinical innate immunity research in mice is hampered by the difficulty of studying human inflammatory reactions in an *in vivo* context. We therefore sought to establish *in vivo* human inflammatory responses in NSG-QUAD mice that express four human myelopoiesis transgenes to improve engraftment of a human innate immune system.

**Methods:**

We reconstituted NSG-QUAD mice with human hematopoietic stem and progenitor cells (HSPCs), after which we evaluated human myeloid cell development and subsequent human responses to systemic and local lipopolysaccharide (LPS) challenges.

**Results:**

NSG-QUAD mice already displayed engraftment of human monocytes, dendritic cells and granulocytes in peripheral blood, spleen and liver at 6 weeks after HSPC reconstitution, in which both classical, intermediate and non-classical monocytes were present. These huNSG-QUAD mice responded to intraperitoneal and intranasal LPS challenges with production of NF-κB-dependent human cytokines, a human type I interferon response, as well as inflammasome-mediated production of human IL-1β and IL-18. The latter were specifically abrogated by the NLRP3 inhibitor MCC950, while LPS-induced human monocyte death was not altered. Besides providing proof-of-principle for small molecule testing of human inflammatory reactions in huNSG-QUAD mice, this observation suggests that LPS-induced *in vivo* release of human NLRP3 inflammasome-generated cytokines occurs in a cell death-independent manner.

**Conclusion:**

HuNSG-QUAD mice are competent for the NF-κB, interferon and inflammasome effectors of human innate immunity, and can thus be utilized to investigate signaling mechanisms and pharmacological targeting of human inflammatory responses in an *in vivo* setting.

## 1 Introduction

The innate immune system plays an essential role in the host response aiming to restore homeostasis upon infection and tissue damage. To this end, innate immune cells employ several classes of pattern recognition receptors (PRRs) that sense pathogen-associated molecular patterns (PAMPs) or danger-associated molecular patterns (DAMPs) to trigger the secretion of inflammatory effector proteins. Myeloid cells such as monocytes, macrophages and dendritic cells (DCs) are particularly competent for these proinflammatory signaling cascades that induce NF-κB-dependent cytokines and type I interferon (IFN)-mediated responses, as well as post-translational inflammasome-mediated cytokine processing (1). While essential for coordinating innate immune responses, hyperactivity of each of these individual NF-κB, type I IFN or inflammasome signaling pathways suffices to provoke autoinflammatory diseases (2–4). Accordingly, dysregulated activities of these innate immune pathways are also involved in a wide variety of more complex inflammation-associated disorders (5).

In the past decades, conventional mouse models have been instrumental in revealing the molecular mechanisms by which innate immune signaling pathways convey detrimental effects in inflammation, thereby suggesting therapeutic approaches for the treatment of human inflammatory diseases. However, despite complementary studies in human cell lines, primary cells or patient samples, translating preclinical innate immunity research in mice toward concrete clinical advances for humans remains hampered by the difficulty to study human inflammatory pathways in an *in vivo* context (6). Transplanting human hematopoietic stem and progenitor cells (HSPCs) into NOD-scid-Il2rγ^-/-^ (NSG) mice can overcome this difficulty, as these immunodeficient NSG mice provide an optimal environment for efficient engraftment of HSPCs and development of a human immune system (7–9). Although these humanized NSG mice significantly advanced translational adaptive immunology, limited species cross-reactivity between key developmental drivers of myeloid cells has proven a hurdle for functional development of human myeloid cells and thereby for enabling *in vivo* human innate immunity research (10, 11). Therefore, to boost human myelopoiesis, NSG-SGM3 mice, which express human stem cell factor (SCF), granulocyte macrophage colony-stimulating factor (GM-CSF) and interleukin (IL)-3, were generated (12). These three human transgenes allowed NSG-SGM3 mice to better engraft various human myeloid cell types including monocytes, DCs, mast cells, neutrophils and other granulocytes (13–16). Using a similar transgenic approach, NSG-CSF1 mice expressing human colony stimulating factor (CSF)1 showed improved human monocyte/macrophage development following a humanization protocol engrafting human bone marrow, fetal liver and thymus (17).

In this study, we used NSG-QUAD mice, derived from intercrossing NSG-SGM3 and NSG-CSF1 lines, which, as such, harbor four human myelopoiesis transgenes to optimize the development of a functional human innate immune system. One study previously showed that NSG-QUAD mice outperform NSG-SGM3 mice in terms of human monocyte engraftment as well as cytokine production (11), suggesting an improved ability to model human innate immunity. However, neither the diversity of the reconstituted human innate immune system nor the preclinical value for targeting inflammatory signaling pathways was previously assessed in humanized NSG-QUAD mice. Here, we characterized the human innate immune system that develops in HSPC-reconstituted NSG-QUAD mice, and we demonstrate its functionality in producing prototypical human NF-κB responsive cytokines, human type I IFN responses, as well as inflammasome-generated human cytokine responses. Lastly, we provide proof-of-concept for pathway-specific therapeutics validation by using the NLRP3 inflammasome inhibitor MCC950 to interfere with human inflammasome responses in NSG-QUAD mice. Thus, we here propose HSPC-reconstituted NSG-QUAD mice as a humanized mouse model suitable for preclinical *in vivo* human innate immunity research.

## 2 Materials and methods

### 2.1 Mice

NOD.Cg-*Prkdc* ^scid^ *Il2rg* ^tm1Wjl^Tg(CMV-IL3,CSF2,KITLG)1EavTg(CSF1)3Sz/J (NSG-QUAD) mice were obtained from Jackson laboratories (RRID: IMSR_JAX:028657, JAX stock #028657) and were maintained in the specific pathogen free facility at VIB-IRC. These NSG-QUAD mice carry homozygous SCF, GM-CSF and IL3 human transgenes, while the human CSF1 transgene was kept hemizygous due to fertility issues in CSF1^Tg^ homozygotes. Colonies were maintained by crossing CSF1 hemizygous NSG-QUAD mice to NSG mice carrying homozygous SCF, GM-CSF and IL3 human transgenes (also known as NSG-SGM3 mice, JAX stock #005557), after which genotyping for the human SCF1 transgene identified quadruple transgene NSG-QUAD mice to be used for humanization experiments. Mice were housed in individually ventilated cages in a 12h-light-12h-dark cycle and were fed standard rodent feed (Ssniff, Soest, Germany) ad libitum with free access to drinking water. Following engraftment with HSPCs, mice were housed in a biosafety level 2 compliant facility. All animal experiments were performed according to institutionally approved protocols according to national (Belgian Laws 14/08/1986 and 22/12/2003, Belgian Royal Decree 06/04/2010) and European (EU Directives 2010/63/EU, 86/609/EEG) animal regulations. Animal protocols were reviewed and approved by the Ethical Committee Animal Experimentation VIB site Ghent - Ghent University - Faculty of Sciences (permit number LA1400091) with approval ID EC2022-003. All necessary efforts were made to minimize suffering of the animals.

### 2.2 Engraftment of human CD34^+^ hematopoietic stem and progenitor cells into NSG-QUAD mice

Human CD34^+^ HSPCs were isolated from umbilical cord blood, which was obtained from the Cord Blood Bank UZ Gent, via density gradient using lymphoprep (StemCell Technologies), followed by positive magnetic-activated cell sorting (MACS) separation using the human CD34 MicroBead Kit (Miltenyi). Cells were either frozen in fetal calf serum (FCS) (TICO International Medicine) supplemented with 10% DMSO (Sigma), or used directly. Both frozen and fresh cells were allowed to recover in cytokine rich medium [StemSpan SFEM II (Stem Cell Technologies) supplemented with 100 ng/ml recombinant human SCF (Peprotech), and 20 ng/ml recombinant human thrombopoietin (TPO) (Peprotech), and 100 ng/ml human Flt3L (VIB-protein core)] overnight prior to engraftment. Only cells containing less than 1% CD3^+^ T cells were used to engraft humanized mice. All human samples were used with permission of and according to the guidelines approved by the Medical Ethical Committee of Ghent University Hospital (BC-06143, February 25, 2020) after informed consent had been obtained in accordance with the Declaration of Helsinki.

To enable human HSPC engraftment, 3-6 weeks old mice were sublethally irradiated with 100-150 cGy (X-ray irradiation, X-RAD 320, Precision X-ray Inc.). Six hours later, mice were intravenously injected with 50 000 – 100 000 human HSPCs, depending on the yield of donor HSPCs and the number of recipient mice in the respective experiment. Within each cohort reconstituted with HSPCs from a particular donor, all recipient mice were age- and sex-matched littermates. HSPC-reconstituted mice were bled 5 weeks post-injection to assess human immune system engraftment and experiments were performed at 6-7 weeks post-injection. Mice that did not surpass the threshold of 10% human CD45^+^ immune cells were considered not successfully humanized and were removed from the experiment. With each cohort of humanized mice, at least one control non-humanized mouse was included in order to confirm the human specificity of all analyses performed.

### 2.3 Flow cytometry analyses and antibodies

Human immune reconstitution was assessed by multicolor flow cytometry. Peripheral blood was collected in K3 EDTA-coated tubes (Sarstedt) from the tail vein at intermediate time points, and via cardiac puncture for end-point analysis. ACK buffer (Lonza) was used to lyse red blood cells. Single cell suspensions of liver and spleen were prepared by mechanical dissociation, cell suspensions were passed through 70 μM cell strainers and ACK buffer (Lonza) was used to lyse the red blood cells. For the liver, immune cells were further isolated using a 37.5% Percoll (cytiva) gradient centrifugation.

The following dyes and antibodies were used for multicolor flow cytometry staining: hCD3#Brilliant Violet 605 (SK7), hCD3#Brilliant Violet 650 (OKT3), hCD19#Brilliant Violet 650 (HIB19) hHLA-A,B,C#APC/Cyanine7 (W6/32), hCD14#PE/Cyanine7 (M5E2), hCD66b#APC (G10F5), mCD45#PE (30-F11) hCD34#APC (581) (Biolegend), (ThermoFisher Scientific), hHLA-DR#Brilliant Ultra Violet 737 (LN3), hCD19#APC (HIB19) (BD Pharmingen), hCD45#BUV395 (HI30) (BD Horizon). To assess human myeloid reconstitution at week 6 post-engraftment, cells were fixed using Foxp3/Transcription factor fixation/permeabilization concentration and diluent (Invitrogen) following the manufacturers protocol. The samples were acquired on a BD LSR Fortessa cytometer (BD) or on a BD LSR II Tubes cytometer (BD) and analysis was carried out using FlowJo v10.

### 2.4 *In vivo* inflammatory challenge experiments

All mice used in *in vivo* experiments were age- and sex-matched littermates. Mice were injected intraperitoneally with 15 μg LPS or intranasally with 6 mg/kg LPS from *E. coli* O111:B4 (Sigma Aldrich, L2630) dissolved in sterile PBS, or intraperitoneally with 200 μg R848 (Invivogen, tlrl-r848-5), and sacrificed 6 hours post-injection. To inhibit the NLRP3 inflammasome mice were injected intraperitoneally with 50 mg/kg MCC950 (MCE MedChemExpress, HY-12815A) or with vehicle 1 hour prior to LPS challenge. Bronchoalveolar lavage (BAL) fluid was collected by flushing the lung four times with 1mL ethylenediaminetetraacetic acid-containing PBS (0.5 mmol/L) via a cannula inserted in the trachea. The first collected 1mL BAL fluid was transferred to a 15 ml falcon tube and the 2^nd^, 3^rd^, and 4^th^ 1mL of BAL fluid was collected in a separate 15 ml falcon tube. BAL fluid cells were pelleted by centrifugation (7 minutes, 400g) for flow cytometry and supernatant of the 1st 1mL was used for cytokine measurements.

### 2.5 Cytokine measurements

Blood was collected by tail vein bleeding or by cardiac puncture and was then kept at room temperature for 30 minutes to clot followed by cold centrifugation for 10 minutes at 2200g to prepare serum for cytokine analyses. Tissue samples were weighed and were homogenized in PBS with protease inhibitors, after which lysis was completed by addition of lysis buffer (20 mM Tris HCl (pH 7.4), 200 mM NaCl, 1% Nonidet P-40) and incubation for 20 minutes on ice. Full speed centrifugation for 30 minutes cleared the homogenate and supernatant was used for further analysis. Human cytokines in serum, BAL, or tissue homogenates were determined by magnetic bead-based multiplex assay using Luminex technology (Bio-Rad, Hercules, CA, USA) according to the manufacturer’s instructions. Serum and BAL cytokines were expressed as concentration per ml of serum or BAL, while cytokines from tissue homogenates were normalized to weight of tissue.

### 2.6 Statistics

In cases where more than one donor was included in an experiment, human cytokine analyses were performed in R. For this, log-transformed cytokine levels were analyzed by a two-way ANOVA, having donor (two levels) as blocking factor. The significance of the main terms treatment and cytokine and their interaction was assessed by an F-test. The significance level for multiple pairwise comparisons between the two treatment levels were adjusted according to Sidak’s multiple comparison test. In cases where only one donor was included in an experiment, analyses were performed in Graphpad Prism 10.1 software. For this, log-transformed cytokine levels were analyzed by an unpaired t test or Mann Whitney U test, depending on the normal distribution of the data. Other analyses were performed in Genstat (version 22, VSN International). For count data, a log-linear regression model, with a log link function, as implemented in Genstat (version 24, VSN International) was fitted to the response data. In both approaches, the donor served as blocking factor in case at least two levels were present. In these analyses, due to the binomial nature of the proportion data, a logistic regression model with a logit link function was fitted to the response data. The dispersion parameter for the variance of the response was estimated from the residual mean square of the fitted model. Wald statistics were used to assess the significance of the main treatments and their interaction terms, by dropping these fixed terms from the full model. T-statistics were used to assess the significance of treatment (e.g. PBS, LPS, LPS + MCC950) effects (on the logit transformed scale) by pairwise comparisons to reference treatment level. Schemes were made using Biorender.

## 3 Results

### 3.1 Human myeloid cells engraft rapidly and efficiently in HSPC-reconstituted NSG-QUAD mice

**Figure 1:**
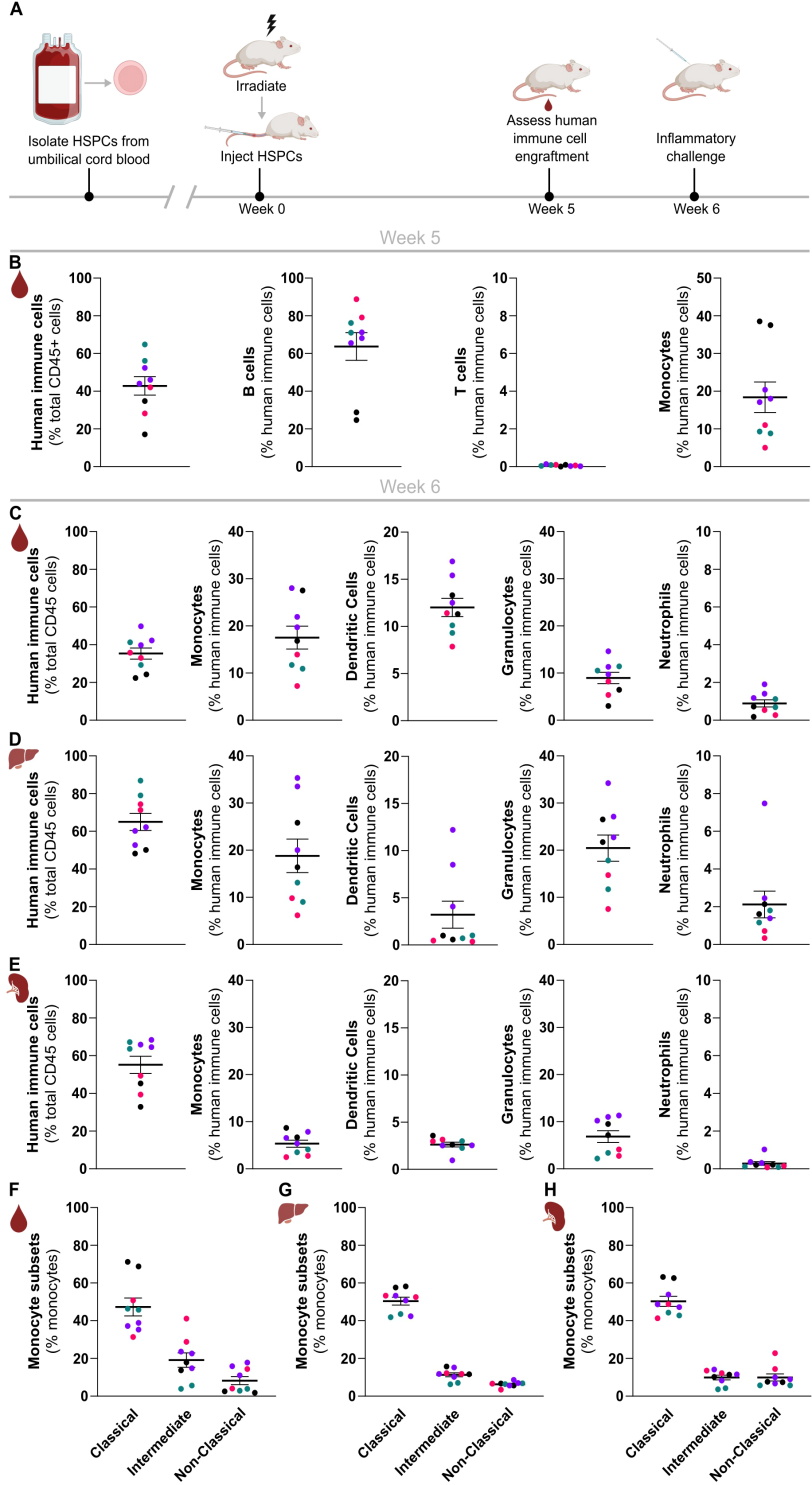
Rapid and efficient reconstitution of a human myeloid immune system in NSG-QUAD mice. **(A)** Schematic overview of the NSG-QUAD humanization and analysis procedures. **(B)** Human immune cell reconstitution in peripheral blood 5 weeks post-engraftment. Human immune cells = hCD45^+^hHLA-ABC^+^, B cells = hCD45^+^hHLA-ABC^+^hCD19^+^, T cells = hCD45^+^hHLA-ABC^+^hCD3^+^, monocytes = hCD45^+^hHLA-ABC^+^hCD14^+^. **(C-E)** Human myeloid reconstitution 6 weeks post-engraftment in **(C)** blood, **(D)** liver, and **(E)** spleen. Human immune cells = hCD45^+^, monocytes = hCD45^+^hCD66b^-^hCD14^+^, dendritic cells = hCD45^+^hCD66b^-^hCD14^-^hCD19^-^hCD3^-^hHLA-DR^+.^ granulocytes = hCD45^+^hCD66b^+^hCD16^-^, and neutrophils = hCD45^+^hCD66b^+^hCD16^+^. **(F–H)** Human monocyte subsets 6 weeks post-engraftment in **(F)** blood, **(G)** liver, and **(H)** spleen respectively. Classical monocytes = hCD14^++^hCD16^-^, intermediate monocytes = hCD14^++^hCD16^+^, and non-classical monocytes = hCD14^+^hCD16^+^. All data represent means ± SEM with dots representing individual mice, and different colors representing different HSPC donors.

To engraft a human immune system in NSG-QUAD mice, we sublethally irradiated these mice and injected them 6 hours later intravenously with HSPCs obtained from human umbilical cord blood via hCD34^+^ cell enrichment (**Figure 1A**). To enable efficient human immune cell engraftments, all donor cells were confirmed to contain at least 85% hCD34^+^ HSPCs and less than 1% hCD3^+^ T cells to avoid rapid graft-versus-host-disease (GvHD). As early as 5 weeks post-engraftment, quantifying human versus murine CD45^+^ cells in peripheral blood demonstrated efficient human immune cell reconstitution, as NSG-QUAD recipients contained 15-65% hCD45^+^ cells depending on both human donor and mouse recipient variability (**Figure 1B**). Multicolor flow cytometry analyses (**Supplementary Figure 1A**) further showed that the majority of peripheral hCD45^+^ immune cells in these humanized NSG-QUAD (huNSG-QUAD) mice were hCD19^+^ B cells, while hCD3^+^ T cells were absent at 5 weeks post-engraftment (**Figure 1B**). This is consistent with prior human T cell development kinetics observations showing that intravenous hCD34^+^ HSPC reconstitution leads to circulating human T cells only in young recipient NSG mice starting around 12 weeks post-engraftment (7, 18). In contrast, 5 weeks of engraftment was sufficient for human monocyte development, as hCD14^+^ cells on average accounted for over 15% of hCD45^+^ cells in peripheral blood of huNSG-QUAD mice, although variation in monocyte engraftment was observed depending on the human cord blood donor (**Figure 1B**). Taken together, these peripheral blood analyses at 5 weeks after HSPC injection demonstrated that huNSG-QUAD mice engrafted a partial human immune system defective for T cells but comprising a proportion of monocytes suggesting a certain degree of innate immune competence.

Therefore, in order to investigate the suitability and applicability of this humanized mouse model for innate immunity research in more detail, we investigated the reconstitution of various types of human myeloid cells in peripheral blood as well as in spleen and liver of these huNSG-QUAD mice at 6 weeks post-engraftment (**Supplementary Figure 1B**). At this timepoint, huNSG-QUAD mice showed similar hCD14^+^ monocyte numbers in peripheral blood as observed at 5 weeks post-engraftment (**Figure 1C**). After monocytes, Lin^-^HLA-DR^+^ DCs were the second most prominent human myeloid cell type in peripheral blood of these mice, accounting for around 12% of hCD45^+^ cells (**Figure 1C**). Finally, while huNSG-QUAD mice contained very few hCD66b^+^hCD16^+^ neutrophils (less than 2% of peripheral hCD45^+^ cells), non-neutrophilic hCD66b^+^hCD16^-^ granulocytes comprised about 10% of their peripheral hCD45^+^ cells (**Figure 1C**). Identical human myeloid cell analyses in liver and spleen of these huNSG-QUAD mice similarly showed hepatic and splenic engraftment of human monocytes, DCs as well as non-neutrophilic granulocytes, while human neutrophils were rare (**Figures 1D, E**). More specifically, the proportions of human myeloid cells in the liver of huNSG-QUAD mice largely reflected those seen in peripheral blood with the exception of DCs, which were present at lower frequencies in the liver than in the blood (**Figure 1D**). Compared to the liver, the spleen of huNSG-QUAD mice overall contained lower proportions of the various human myeloid cell types despite engrafting similar amounts of hCD45^+^ cells (**Figure 1E**), most probably due to the occupation of the splenic niche by the abundant hCD19^+^ B cells in these mice. Thus, these immunophenotyping analyses revealed engraftment of various human innate immune cells in blood, liver and spleen of huNSG-QUAD mice, with hCD14^+^ monocytes emerging as the predominant human myeloid cell type. As human monocytes are classified into three functionally distinct subsets based on their CD14 and CD16 expression levels, we next evaluated the subset identities of the engrafted hCD14^+^ cells (**Supplementary Figure 1B**). This showed that hCD14^+^ cells in blood, liver as well as spleen of huNSG-QUAD mice mainly comprised CD14^++^CD16^-^ classical monocytes, although CD14^++^CD16^+^ intermediate monocytes and CD14^+^CD16^+^ non-classical monocytes were also present (**Figures 1F–H**). In conclusion, NSG-QUAD mice allow the development of a diverse human myeloid cell spectrum comprising all three subsets of human monocytes, as well as DCs and granulocytes already at 6 weeks after HPSC reconstitution, suggesting a potential value for huNSG-QUAD mice for innate immunity research. More advanced human immune system engraftment in huNSG-QUAD mice at later timepoints was not evaluated since we observed weight loss and mortality in a number of huNSG-QUAD mice when kept beyond 9 weeks post-humanization (data not shown).

### 3.2 HuNSG-QUAD mice are competent to induce the NF-κB-, type I IFN- and inflammasome-controlled arms of systemic human innate immune responses

**Figure 2:**
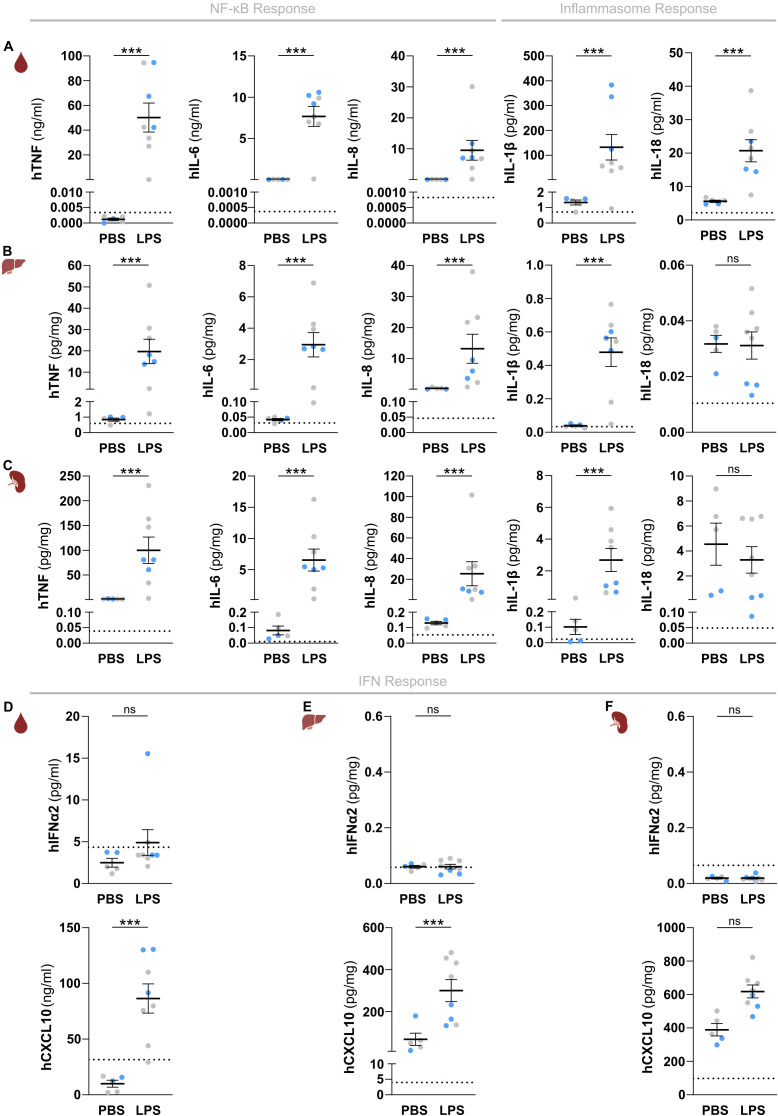
HuNSG-QUAD mice display human NF-κB and inflammasome cytokine responses as well as a human type I IFN response upon a systemic LPS challenge. **(A–C)** Sex-matched huNSG-QUAD littermates were injected intraperitoneally with PBS or with 15 μg LPS at 6 weeks post-engraftment. Indicated human NF-κB and inflammasome-dependent cytokines were measured in **(A)** serum, **(B)** liver, and **(C)** spleen 6 hours after the challenge. **(D–F)** Human IFNα2 and CXCL10 were measured in **(D)** serum, **(E)** liver, and **(F)** spleen 6 hours after the challenge. All data represent means ± SEM with dots representing individual mice, and different colors representing different HSPC donors. The dotted line on each graph represents the murine background level of the respective ‘human protein’ detected in respective samples from an LPS-injected non-humanized NSG-QUAD mouse using this assay. Statistics were analyzed by a two-way ANOVA on log-transformed data followed by Sidak’s multiple comparison tests. ***p<0.001; ns not significant.

Having established that huNSG-QUAD mice already host various human myeloid cell types at 6 weeks post-engraftment, we next evaluated the ability of these mice to provoke human innate immune responses at this stage. To do so, we first applied a model of systemic endotoxemia by injecting huNSG-QUAD mice intraperitoneally with lipopolysaccharide (LPS). Upon engaging TLR4, LPS induces divergent signaling pathways controlling innate immunity. A MyD88-dependent pathway leads to the expression of NF-κB-dependent cytokines, whereas a TRIF-dependent pathway provokes type I IFNs and a downstream IFN-stimulated gene (ISG) response. In addition, LPS is known to induce secretion of human IL-1β and IL-18 by activating an inflammasome complex that proteolytically matures the pro-forms of these cytokines to their bio-active forms. We therefore assessed all three of these responses by measuring LPS-induced production of the prototypical NF-κB-dependent factors TNF, IL-6 and IL-8; the inflammasome-generated cytokines IL-1β and IL-18; as well as IFNα2 and CXCL10 as, respectively, a type I IFN and a chemokine expressed downstream of type I IFNs in human myeloid cells (19, 20). At 6 hours post-LPS injection, huNSG-QUAD mice displayed significantly elevated levels of human TNF, IL-6 and IL-8 in their serum, liver and spleen as compared to PBS-injected huNSG-QUAD mice (**Figures 2A–C**). As pro-IL-1β requires NF-κB-dependent transcriptional upregulation prior to its inflammasome-mediated processing, these functional LPS-induced human NF-κB responses in huNSG-QUAD mice suggested that these mice could harbor sufficient human pro-IL-1β levels to enable the detection of mature hIL-1β. Indeed, at 6 hours after intraperitoneal LPS challenge, huNSG-QUAD mice displayed elevated serum levels of hIL-1β and hIL-18 as compared to PBS-challenged littermates (**Figure 2A**). In line with this observation, livers and spleens of intraperitoneal LPS-injected huNSG-QUAD mice also showed increased hIL-1β levels, albeit at much lower concentrations than in serum and not accompanied by increased human IL-18 levels (**Figures 2B, C**). These human IL-1β and IL-18 measurements demonstrate that huNSG-QUAD mice harbor functional human inflammasome complexes capable of driving secretion of these cytokines, of which hIL-1β appears to represent the most sensitive marker for detecting human inflammasome activity in these mice. In contrast to these NF-κB and inflammasome responses, LPS-challenged huNSG-QUAD mice did not show an obvious type I IFN response. Indeed, human IFNα2 levels did not even surpass the background signals detected in samples from an LPS-injected non-humanized NSG-QUAD mouse that served as control in all measurements to ensure human specificity of the applied immunoassays (**Figures 2D–F**). Nevertheless, human CXCL10 was upregulated in serum and liver of LPS-injected huNSG-QUAD mice (**Figures 2D–F**), indicating that these mice to some extent did respond to LPS with a human ISG response.

To characterize the kinetics of the observed human innate immune responses in huNSG-QUAD mice we measured cytokines at 1, 3 and 6 hours after an intraperitoneal LPS injection. These analyses showed that NF-κB- and inflammasome-dependent human cytokine responses were already detectable at 1 and 3 hours post-LPS, respectively (**Supplementary Figures 2A, B**). Interestingly, human IFNα2 serum levels showed a transient upregulation at 3 hours post-LPS, while human CXCL10 was only detectable in the serum 6 hours after the LPS challenge (**Supplementary Figure 2C**). This observation is in line with a typical type I IFN response in which LPS-induced hIFNα2 subsequently triggers expression of ISGs such as hCXCL10, and may explain the lack of hIFNα2 in our earlier snapshot analyses at 6 hours post-LPS. To further support the ability of huNSG-QUAD mice to elicit a human type I IFN response, we subjected these mice to an intraperitoneal injection with resiquimod (R848) to stimulate the TLR7/8 receptors that more selectively activate ISG responses. Indeed, besides inducing lower levels of human NF-κB-dependent cytokines when compared to LPS, R848 was able to provoke elevated levels of both hIFNα2 and hCXCL10 in serum and spleen of huNSG-QUAD mice (**Supplementary Figure 3**). Of note, systemic R848 administration did not elevate inflammasome-generated serum cytokines, although minor hIL-1β upregulations were observed in livers and spleens of R848-injected huNSG-QUAD mice (**Supplementary Figure 4**). These differential effects of LPS and R848 may reflect different signaling mechanisms or different cellular sources of human inflammasome responses in huNSG-QUAD mice.

Taken together, our analyses measuring several inflammatory mediators upon TLR4 or TLR7/8 triggering revealed that huNSG-QUAD mice are competent for three major drivers of human innate immune responses. Functional human NF-κB, type I IFN, and inflammasome signaling pathways in huNSG-QUAD mice facilitated LPS-induced production of various human innate immunity effectors.

### 3.3 HuNSG-QUAD mice are competent to induce the NF-κB-, type I IFN- and inflammasome-controlled arms of pulmonary human innate immune responses

**Figure 3:**
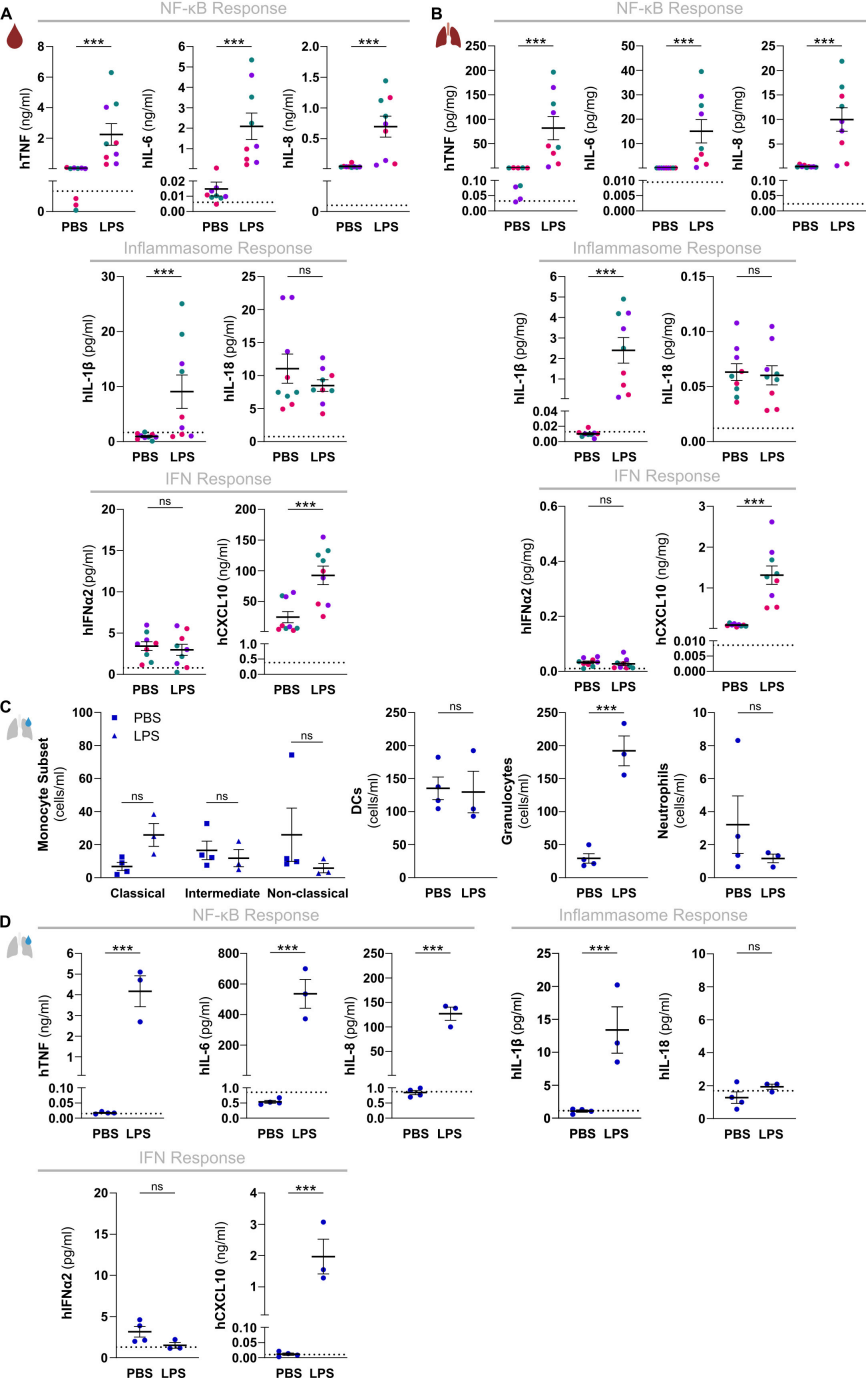
HuNSG-QUAD mice display human NF-κB and inflammasome cytokine responses as well as a human type I IFN response upon a local LPS challenge. Sex-matched huNSG-QUAD littermates were injected intranasally with PBS or with 6 mg/kg LPS at 6 weeks post-engraftment. **(A, B)** Indicated human proteins were measured in **(A)** serum and **(B)** lungs 6 hours after the challenge. **(C, D)** BAL fluid was collected 6 hours after the challenge, in which **(C)** the indicated human myeloid cells (classical monocytes = hCD45^+^hCD14^++^hCD16^-^, intermediate monocytes = hCD45^+^hCD14^++^hCD16^+^, non-classical monocytes = hCD45^+^hCD14^+^hCD16^+^, DCs = hCD45^+^hCD66b^-^hCD14^-^hCD19^-^hCD3^-^hHLA-DR^+^, granulocytes = hCD45^+^hCD66b^+^hCD16^-^, and neutrophils = hCD45^+^hCD66b^+^hCD16^+^) were quantified via flow cytometry, and **(D)** indicated human proteins were measured. All data represent means ± SEM with dots representing individual mice, and different colors representing different HPSC donors. The dotted lines in A, B and D represent the murine background level of the respective ‘human protein’ detected in respective samples from an LPS-injected non-humanized NSG-QUAD mouse using this assay. Statistics in **(A, B)** were analyzed by a two-way ANOVA on log-transformed data followed by Sidak’s multiple comparison tests. Statistics in **(C)** were analyzed using Wald and T statistics applied on a linear regression model, as described in the methods. Statistics in **(D)** were analyzed by an unpaired T test or Mann Whitney tests on log-transformed data. ***p<0.001; ns not significant.

We next examined the functionality of these three signaling hubs controlling innate immune responses in a local organ-specific inflammatory model by administering LPS intranasally to huNSG-QUAD mice. As observed upon a systemic challenge, intranasal LPS increased hTNF, hIL-6, hIL-8, hIL-1β and hCXCL10 levels in circulation of huNSG-QUAD mice, albeit with lower concentrations as compared to an intraperitoneal LPS injection (**Figure 3A**). In contrast, hIFNα2 and hIL-18 serum levels remained unaffected (**Figure 3A**). Importantly however, lung homogenates displayed a similar pattern of upregulated human cytokines and chemokines upon intranasal LPS challenge (**Figure 3B**), indicating that local pulmonary human innate immune responses can be measured in these mice. To assess the intranasal LPS-induced inflammatory response of huNSG-QUAD mice in more detail we collected BAL fluid to evaluate infiltration of various human innate immune cells. At 6 hours after intranasal LPS challenge, none of the three monocyte subtypes, nor DCs or neutrophils had infiltrated the BAL of huNSG-QUAD mice (**Figure 3C**). However, BAL fluid of huNSG-QUAD mice contained a significant increase in non-neutrophilic human granulocytes at this timepoint (**Figure 3C**). In addition, cytokine measurements in these BAL fluids revealed intranasal LPS-induced elevations in human TNF, IL-6, IL-8, IL-1β and CXCL10 levels (**Figure 3D**), reflecting earlier findings in lung homogenates. These observations suggest that intranasal LPS induces a cellular response with actively infiltrating as well as cytokine producing human myeloid cells in the lungs of huNSG-QUAD mice.

Thus, our intraperitoneal and intranasal LPS challenge experiments together show that huNSG-QUAD mice are capable of eliciting the human NF-κB-, inflammasome- and ISG-dependent steering mechanisms of innate immunity in response to both systemic and local LPS challenges. The detectable outputs of each of these three human signaling pathways important in innate immunity collectively suggest that huNSG-QUAD mice may serve as a platform to study the mechanisms of human inflammatory diseases in an experimental *in vivo* context.

### 3.4 HuNSG-QUAD mice are suitable for evaluating *in vivo* pharmacological interference with human NLRP3 inflammasome-mediated innate immune responses

**Figure 4:**
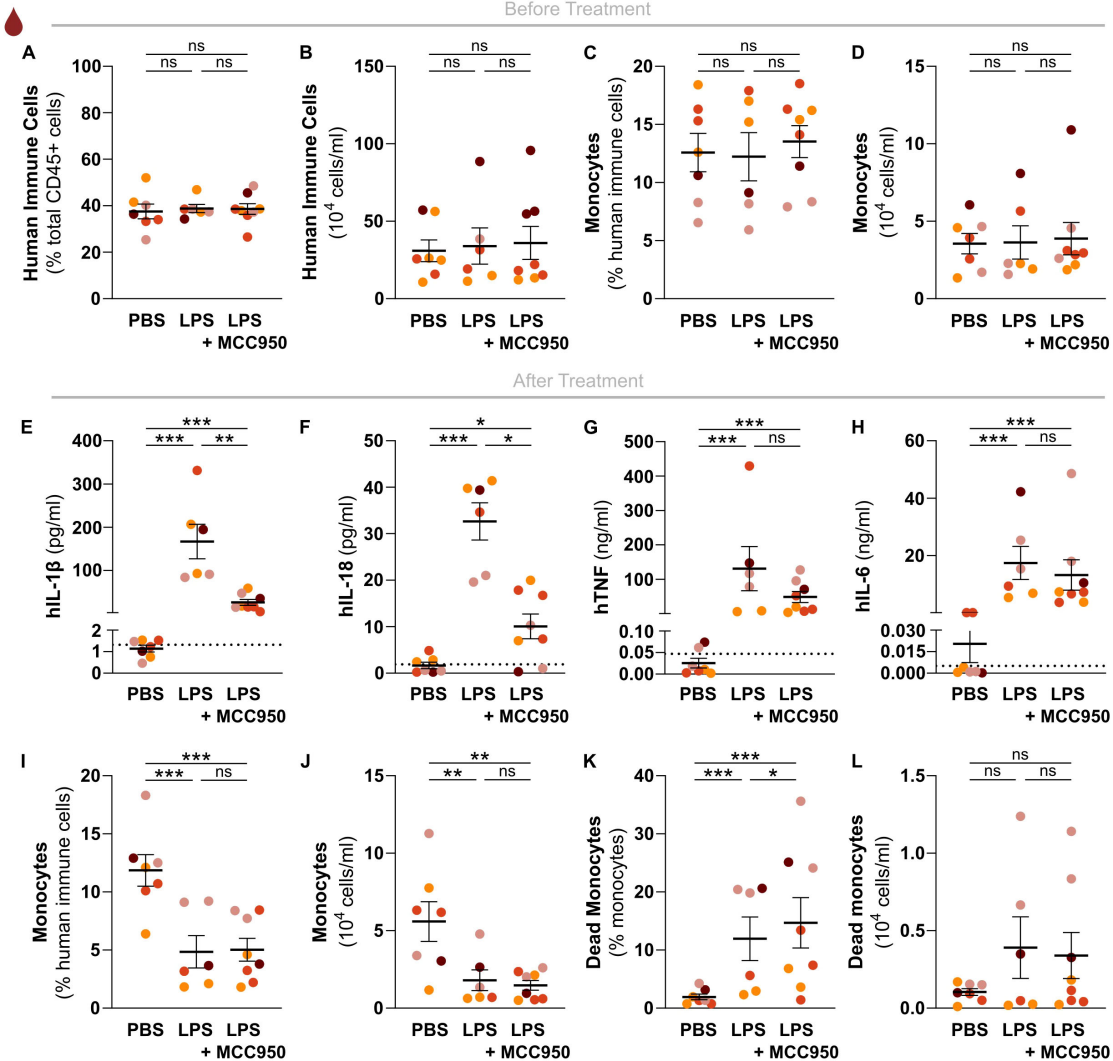
The human NLRP3 inflammasome mediates *in vivo* LPS-induced hIL-1β and hIL-18 production independently of hCD14^+^ monocyte cell death. Relative and absolute numbers of **(A, B)** human immune cells, and of **(C, D)** human monocytes in peripheral blood of sex-matched huNSG-QUAD littermates randomized into the indicated experimental cohorts at 5 weeks post-engraftment. Human immune cells = hCD45^+^, monocytes = hCD45^+^hCD14^+^. **(E–H)** At 6 weeks post-engraftment, huNSG-QUAD cohorts were injected intraperitoneally with either vehicle or 50 mg/kg MCC950, and injected intraperitoneally 1 hour later with PBS or with 15 μg LPS as indicated. Inflammasome-generated **(E)** hIL-1β and **(F)** hIL-18 cytokines, and inflammasome-independent **(G)** hTNF and **(H)** hIL-6 cytokines were measured in serum 6 hours after PBS or LPS challenge. **(I–L)** At 6 hours after PBS or LPS challenge, relative and absolute numbers of **(I, J)** human monocytes, as well as of **(K, L)** dead human monocytes were measured in peripheral blood of the respective huNSG-QUAD cohorts. Human monocytes = hCD45^+^hCD14^+^. All data represent means ± SEM with dots representing individual mice, and different colors representing different HPSC donors. The dotted lines in E-H represent the murine background level of the respective ‘human protein’ detected in respective samples from an LPS-injected non-humanized NSG-QUAD mouse using this assay. For the cytokine data, statistics were analyzed by a two-way ANOVA on log-transformed data followed by Sidak’s multiple comparison tests. For the cell data, Wald and T statistics were applied on a linear regression model, as described in the methods. *p<0.05; **p<0.01; ***p<0.001; ns not significant.

Given the importance of hIL-1β in driving numerous inflammation-associated diseases, inflammasomes are attractive drug targets. In particular the NLRP3 inflammasome provokes hIL-1β release upon a wide variety of inflammatory triggers, which sparked the development of multiple pharmacological NLRP3 inhibitors that are being tested for potential future clinical use (21). Since the NLRP3 inflammasome also mediates LPS-induced release of hIL-1β, we used the pharmacological NLRP3 inhibitor MCC950 (22) to test whether the endotoxemia model in huNSG-QUAD mice can be used to assess the efficacy and specificity of *in vivo* human NLRP3 inflammasome targeting. One week before the experiment, we randomized mice into a PBS control cohort, an LPS challenge cohort and an MCC950-treated LPS challenge cohort, and we verified that all three cohorts showed comparable hCD45^+^ humanization rates (**Figures 4A, B**). Moreover, we confirmed that these cohorts displayed similar proportions as well as absolute numbers of peripheral hCD14+ monocytes (**Figures 4C, D**). These observations minimized potential confounding effects of varying monocyte engraftment rates on LPS-induced hIL-1β production, as monocytes are major hIL-1β producers in response to LPS (23). At 6 hours after intraperitoneal LPS injection, MCC950-treated huNSG-QUAD mice displayed significantly less serum hIL-1β and hIL-18 levels than vehicle-treated LPS-challenged huNSG-QUAD mice (**Figures 4E, F**). In contrast, the minor upregulations of hIL-1β in liver and spleen of LPS-challenged huNSG-QUAD mice were not diminished by MCC950 (**Supplementary Figure 5**). This could reflect an inadequate NLRP3-inhibitory efficiency of MCC950 in liver and spleen, although the lack of effect of blocking the inflammasome could also indicate that the low concentrations of hIL-1β in liver and spleen represented the unprocessed pro-forms of this cytokine. Importantly however, in contrast to hIL-1β and hIL-18 serum levels, hTNF and hIL-6 levels were equally upregulated in serum of LPS-injected huNSG-QUAD mice irrespective of MCC950 treatment (**Figures 4G, H**). Thus, these serum cytokine observations indicated an impact of MCC950 on human inflammasome signaling in huNSG-QUAD mice while leaving inflammasome-independent cytokines unaffected, suggesting that huNSG-QUAD mice are an appropriate model for *in vivo* testing of human NLRP3 inflammasome targeting.

Finally, to better understand the mechanisms underlying *in vivo* human IL-1β production upon LPS challenge, we investigated how LPS-induced monocyte cell death in huNSG-QUAD mice correlated with hIL-1β serum release. Interestingly, in contrast to the similar monocyte numbers in the respective naïve cohorts, LPS-challenged huNSG-QUAD mice showed a reduction in relative as well as absolute hCD14^+^ monocyte numbers in their blood when compared to PBS-injected animals (**Figures 4I, J**). In addition, LPS-treated huNSG-QUAD mice showed more hCD14^+^ monocytes displaying disrupted cellular membranes indicative of ongoing cell death (**Figures 4K, L**; **Supplementary Figure 6**). However, the observed HSPC donor variability in hCD14^+^ cell death rate (**Figures 4K, L**) did not correlate with the variability in serum hIL-1β levels in LPS-challenged huNSG-QUAD mice (**Figure 4E**). Moreover, the reduced hIL-1β levels in MCC950-treated LPS-challenged huNSG-QUAD mice (**Figure 4E**) were not accompanied with a reduction in hCD14^+^ cell death (**Figures 4K, L**). Thus, LPS-induced hIL-1β serum levels in huNSG-QUAD mice did not correlate with cell death of peripheral hCD14^+^ monocytes, arguing that the *in vivo* NLRP3-mediated release of hIL-1β from these cells happens in a cell death-independent manner. This notion is consistent with *in vitro* observations showing that LPS-stimulated human monocytes utilize NLRP3 inflammasome activation pathways leading to hIL-1β secretion independent of cell death induction (24–26). Taken together, we showed proof-of-principle that huNSG-QUAD mice can be used for validating *in vivo* pharmacological agents targeting human NLRP3 inflammasome signaling. In addition, we provided evidence suggesting that cell death-independent NLRP3 pathways previously observed in cultured human monocytes (24–26) are also responsible for LPS-induced human IL-1β release in an *in vivo* context.

## 4 Discussion

Despite the undeniable impact murine studies have had on immunology research, significant species differences often hinder the translation of murine data into successful clinical results for human disease. Humanized mice have been instrumental for closing this species gap especially for studying immuno-oncology and species-restricted infections such as HIV (27, 28), but only few of these mouse models efficiently engraft human myeloid cells to enable studying *in vivo* human inflammatory disease mechanisms. In this study, based on efficient engraftment of various human myeloid cell types and their capacity to drive distinct inflammatory signaling pathways toward cytokine production, we propose HSPC reconstitution of NSG-QUAD mice as a humanized mouse model suitable for studying human innate immunity in an *in vivo* context.

We showed that NSG-QUAD mice already displayed human monocytes, DCs, and neutrophilic as well as non-neutrophilic granulocytes in peripheral blood at 6 weeks after engraftment. Importantly, this rapid human myeloid cell development was achieved after intravenous HSPC reconstitution of adult recipient mice, which is technically less challenging than newborn reconstitutions that are often needed for efficient engraftment of particular human immune cells in immunodeficient recipients. Indeed, limited development of human myeloid cells was already demonstrated in first-generation NSG mice, albeit only 4-6 months after intravenous HSPC reconstitution of newborns (29). Next-generation NSG-SGM3 mice with transgenic complementation of three human myelopoiesis factors showed improved engraftment of a variety of human myeloid cell types, not only upon intrahepatic reconstitution of newborns (13, 15), but also in adult recipients as performed in this NSG-QUAD study (14, 16). Next to NSG-SGM3 mice, so-called MISTRG mice, which harbor transgenes encoding human CSF1, GM-CSF, IL-3, thrombopoietin (TPO) and signal regulatory protein (SIRP)α, also enable efficient human innate immune system engraftment upon intrahepatic HSPC reconstitution of newborns (30, 31). In fact, a side-by-side comparison of human myeloid cell development in NSG-SGM3 and MISTRG mice showed that the latter were superior in terms of human monocyte numbers and CD14/16 diversity (32). In physiological conditions, human blood-circulating monocytes comprise around 85% CD14hiCD16- classical monocytes whereas CD14^hi^CD16^+^ intermediate and CD14^dim^CD16^+^ non-classical monocytes together make up the remaining 15% (33). Indeed, among these subtypes, non-classical human monocytes do not reconstitute in adult NSG-SGM3 mice (14). However, adding the human CSF1 transgene in NSG-QUAD mice increased hCD14^+^ monocyte engraftment as compared to NSG-SGM3 mice (30), and our data showed development of all three human monocyte subtypes in huNSG-QUAD mice in relative quantities nearing the pattern in human blood. As each monocyte subset performs characteristic functions and has its own lifespan (34), a balanced representation of monocyte subsets as in huNSG-QUAD mice might be required to faithfully recapitulate the inflammatory reactions following their activation (33, 35). Ultimately, reconstituting the different immunodeficient mouse models with HSPCs from the same donor via the same route will be needed for a valid side-by-side comparison of human innate immune responses. Nevertheless, our observations place NSG-QUAD mice next to NSG-SGM3 and MISTRG mice as an additional mouse model allowing multilineage human myeloid cell development to model human innate immune responses. Future research will be needed to select the most optimal humanized mouse model for *in vivo* investigation of different human inflammatory diseases depending on their distinct cellular and molecular foundations.

While the rapid development of functional human myeloid cells in adult huNSG-QUAD mice enabled innate immunity challenges already at 6 weeks post-engraftment, a limitation associated with this approach is the lack of human T cell development at this time point. Human T cells develop better in younger immunodeficient recipient mice. For instance, intravenous HSPC administration to adult NSG mice did not allow human T cell engraftment (7), while an identical administration in 3-week-old NSG recipients and even more so an intracardial HSPC injection in newborn NSG mice did provoke peripheral human T cells (18). This age-dependent human T cell development is even more obvious in NSG-SGM3 recipients, as intrahepatic HSPC reconstitution of NSG-SGM3 newborns lead to efficient human T cell differentiation, while an identical approach in 2-day-old NSG-SGM3 mice almost completely abolished this effect (36). These observations suggest that engrafting HSPCs into newborns might also allow human T cell development in NSG-QUAD mice. However, the long-term engraftment periods needed for human T cell development entail a risk for adverse effects in mice with high expression levels of human myelopoiesis factors. As a consequence, huNSG-SGM3 mice show constitutive mobilization of stem cells leading to hematopoietic progenitor cell exhaustion (13). In addition, long-term human myeloid cell engraftment in MISTRG mice provoked destruction of red blood cells leading to anemia-associated death of these humanized mice at 10-12 weeks post-reconstitution (30). Accordingly, and most likely due to similar causes, we observed that some huNSG-QUAD mice lost weight and failed to thrive when kept beyond 9 weeks post-engraftment (data not shown). For this reason, all huNSG-QUAD challenge experiments were carried out at week 6 post-engraftment. An opportunity for optimization could be to use less HSPC donor cells in non-irradiated recipient NSG-QUAD mice, as this was shown to improve long-term post-engraftment survival in MISTRG mice (30). In addition, performing engraftments in NSG-QUAD mice with hemizygous SGM3 transgenes could potentially diminish hematopoietic progenitor cell exhaustion. However, at the moment, the huNSG-QUAD model as described here is only suitable for short-term innate immune challenges while measuring T cell independent readouts.

To induce a short-term innate immune response, we challenged huNSG-QUAD mice systemically and locally with LPS. Both of these challenges provoked clear NF-κB and inflammasome responses, as evidenced by elevated levels of cytokines typically induced by these pathways. In particular, an intraperitoneal LPS injection in huNSG-QUAD mice resulted in similar serum levels of human TNF, IL-6, IL-8 and IL-1β as reported previously in an analogous systemic endotoxemia experiment (11). In addition, huNSG-QUAD mice showed an increase of serum hIFNα2 3 hours after intraperitoneal LPS challenge, followed by elevated hCXCL10 levels three hours later. Since endogenous IFNα/β was shown to mediate hCXCL10 induction in LPS-stimulated primary human monocytes (37), these observations suggested that huNSG-QUAD mice recapitulate the kinetic-specific nuances of a bona fide human type I IFN response and could therefore be used to investigate this further in the future. However, the transient detection of serum hIFNα2 at 3 hours after intraperitoneal LPS in huNSG-QUAD mice illustrates a drawback of the snapshot cytokine analyses at 6 hours as performed in multiple other experiments in this study. For instance, it is possible that huNSG-QUAD mice produce hIL-18 or hIFNα2 at early or at very late timepoints after an intranasal LPS challenge, thereby explaining their lack of detection after 6 hours. Therefore, future kinetic cytokine analyses will be needed to more comprehensively map human innate immune pathway activities in huNSG-QUAD mice. In addition, it should be mentioned that huNSG-QUAD mice harbor murine non-hematopoietic cells as well as some residual murine immune cells that are LPS-responsive. Septic shock experiments in naïve versus humanized NSG mice showed that human cytokines were required to provoke mortality (38), suggesting an inferior role for cytokines produced by the murine immunodeficient host during a systemic innate immune response. However, it is likely that a number of LPS-induced murine cytokines and myelopoietic factors that can bind to their human counterpart receptors contribute to some extent to the human innate immune reactions observed in huNSG-QUAD mice. Future experiments either using species-selective TLR4 agonists (39) or using huNSG-QUAD mice with an additional *Tlr4* deficiency (40) could partially or fully avoid these potential confounding effects of murine cytokines during human innate immune responses in huNSG-QUAD mice. Nevertheless, we believe our current TLR challenge experiments clearly demonstrate that the engrafted human myeloid cells in huNSG-QUAD mice collectively are functionally competent to initiate type I IFN, NF-κB and inflammasome signaling to coordinate a versatile human inflammatory response.

With regards to the human inflammasome response, we investigated whether huNSG-QUAD mice could serve as a platform for *in vivo* validation of inflammasome-targeting compounds and whether this could reveal some of the mechanisms by which human inflammasomes drive *in vivo* cytokine production. We demonstrated that the small molecule NLRP3 inhibitor MCC950, as previously shown in *ex vivo* human PBMCs (22), specifically suppressed the secretion of IL-1β and IL-18 in serum of LPS-challenged huNSG-QUAD mice. As the most abundant human myeloid cells in the blood of huNSG-QUAD mice, monocytes most likely are a main source of these inflammasome-generated cytokines, as observed in humans displaying NLRP3 inflammasome hyperactivation (23). *In vitro* studies showed that human monocytes are competent for two distinct NLRP3 inflammasome pathways upon single LPS treatment. Primary human monocytes were shown to activate a caspase-4/5-driven non-canonical NLRP3 inflammasome pathway upon LPS (26), while LPS experiments in transdifferentiated monocytes demonstrated the existence of a caspase-8-mediated alternative NLRP3 inflammasome pathway (24, 25). However, IL-1β secretion following neither of these pathways was associated with monocyte cell death (24–26). This feature distinguishes these pathways from the canonical NLRP3 inflammasome pathway that requires an activation trigger after LPS administration and that results in pyroptosis (41). In this respect, in agreement with observations in human endotoxemia volunteers (34), we observed human monocyte depletion in huNSG-QUAD mice upon LPS injection. Monocytopenia in these mice could partially, or even fully, be caused by extravasation of monocytes upon intraperitoneal LPS administration. However, for two HSPC donors, LPS-induced monocytopenia in the resulting huNSG-QUAD mice was associated with human monocyte cell death. As opposed to controlled *in vitro* conditions, *in vivo* LPS-induced endotoxemia could be accompanied with the presence of several stimuli combining with LPS to activate the canonical NLRP3 inflammasome leading to cell death. Moreover, endotoxemia studies in human volunteers showed that *in vivo* human monocyte responses do not necessarily reflect their *ex vivo* behavior (42). However, the levels of human monocyte cell death in huNSG-QUAD mice did not correlate with serum IL-1β levels, as efficient blockage of IL-1β production by MCC950 was not accompanied by diminished human monocyte death. These observations indicate that the *in vivo* release of human IL-1β happens independently of monocyte cell death, supporting the involvement of the non-canonical and/or alternative NLRP3 pathways rather than the cell death-associated canonical NLRP3 pathway. Furthermore, since we observed MCC950 inhibition of LPS-induced hIL-18 in serum of LPS-challenged huNSG-QUAD mice, the recent finding that THP-1 monocytes release hIL-18 in an NLRP3-independent manner following non-canonical inflammasome activation (43) suggests that *in vivo* LPS-induced hIL-18 release occurs through the alternative NLRP3-dependent pathway. Alternatively, in contrast to IL-1β, IL-18 may derive from non-monocytic human myeloid cells in LPS-treated huNSG-QUAD mice. Further molecular research in this humanized mouse model will be needed to fully disentangle the cell type-specific pathways controlling the *in vivo* activation of the human NLRP3 inflammasome.

Taken together, we show that huNSG-QUAD mice display versatile systemic human innate immune responses upon LPS administration. These observations already indicate that huNSG-QUAD mice can serve as a translational preclinical model for bacterial sepsis, but future applications can be envisaged. For instance, further establishment of the observed LPS-induced human pulmonary innate immune responses may enable investigation of allergic respiratory disorders and preclinical evaluation of mucosal vaccines. In addition, NSG-QUAD mice were shown to represent better hosts than NSG-SGM3 mice for differentiation of human microglia precursors transplanted into their neonatal brain (44). As such, combining transplantation of human microglia precursors with HPSC-mediated humanization may create huNSG-QUAD mice carrying a more body-wide human innate immune system. In the future, a further thorough characterization of the huNSG-QUAD model will reveal its strengths as well as its weaknesses, which ultimately will be required to appreciate how this preclinical humanized mouse model can be used to effectively lead to new innate immunity insights and to permit the assessment of new anti-inflammatory therapies.

## Data Availability

The original contributions presented in the study are included in the article/**Supplementary Material**. Further inquiries can be directed to the corresponding author.

## Supplementary material

Supplementary Figure 1 Flow cytometric analysis of human immune cells in HSPC-reconstituted NSG-QUAD mice. **(A)** Representative flow-cytometric gating to assess human immune cell reconstitution in peripheral blood at week 5 post-engraftment (as in **Figure 1B**). Doublets and dead cells were excluded before identifying live cells. From total mCD45^+^ and hCD45^+^ cells, human immune cells were identified as hCD45^+^hHLA^+^ cells. From this latter population, human B cells were identified as hCD19^+^ cells, human T cells were identified as hCD3^+^ cells, and human monocytes were identified as hCD14^+^ cells. SSC, side scatter. **(B)** Representative flow-cytometric gating to assess human myeloid immune cell reconstitution in peripheral blood at week 6 post-engraftment (as in **Figures 1C–H**). Doublets and dead cells were excluded before identifying live cells. From total mCD45^+^ and hCD45^+^ cells, human immune cells were identified as hCD45^+^ cells. From this latter population, the hCD66^+^ fraction was further identified as hCD16^+^ neutrophils and hCD16^-^ granulocytes; while the hCD66^-^ fraction was further identified as hCD14^+^ monocytes that were classified into different subtypes based on hCD14 and hCD16 expression levels. From the remaining hCD45^+^hCD66^-^hCD14^-^ fraction, hCD19^+^ B and hCD3^+^ T cells were excluded, after which hHLA-DR^+^ cells were identified as dendritic cells. SSC, side scatter.

Supplementary Figure 2 Kinetics of human NF-κB- and inflammasome-dependent cytokine responses as well as human type I IFN responses in huNSG-QUAD mice upon a systemic LPS challenge. **(A–C)** Sex- and age-matched huNSG-QUAD littermates were injected intraperitoneally with 15 μg LPS at 6 weeks post-engraftment. Blood was collected before the challenge as well as after 1, 3 and 6 hours. Indicated human **(A)** NF-κB-dependent cytokines, **(B)** inflammasome-dependent cytokines, and **(C)** type I IFN response indicators were measured in serum. All data represent means ± SEM with dots representing individual mice reconstituted with HSPCs from the same donor. The dotted line on each graph represents the murine background level of the respective ‘human protein’ detected in serum samples from an LPS-injected non-humanized NSG-QUAD mouse using this assay. Statistics were analyzed by an unpaired t test or Mann Whitney test on log-transformed data. * p<0.05; ** p<0.01; *** p<0.001; ns not significant.

Supplementary Figure 3 HuNSG-QUAD mice display human NF-κB cytokine and type I IFN responses upon a systemic R848 challenge. **(A–C)** Sex- and age-matched huNSG-QUAD littermates were injected intraperitoneally with vehicle or with 200 μg R848. Indicated human proteins were measured in **(A)** serum, **(B)** liver, and **(C)** spleen 6 hours after the challenge. All data represent means ± SEM with dots representing individual mice, and different colors representing different HPSC donors. The dotted line on each graph represents the murine background level of the respective ‘human protein’ detected in respective samples from an R848-injected non-humanized NSG-QUAD mouse using this assay. Statistics were analyzed by a two-way ANOVA on log-transformed data followed by Sidak’s multiple comparison tests. *** p<0.001; ns not significant.

Supplementary Figure 4 HuNSG-QUAD mice display limited human inflammasome responses upon a systemic R848 challenge. **(A–C)** Sex- and age-matched hu-NSG-QUAD littermates were injected intraperitoneally with vehicle or with 200 μg R848. Inflammasome-generated hIL-1β and hIL-18 were measured in **(A)** serum, **(B)** liver, and **(C)** spleen 6 hours after the challenge. All data represent means ± SEM with dots representing individual mice, and different colors representing different HPSC donors. The dotted line on each graph represents the murine background level of the respective ‘human protein’ detected in respective samples from an R848-injected non-humanized NSG-QUAD mouse using this assay. Statistics were analyzed by a two-way ANOVA on log-transformed data followed by Sidak’s multiple comparison tests. *** p<0.001; ns not significant.

Supplementary Figure 5 Splenic and hepatic hIL-1β production is not affected by MCC950 treatment in LPS-challenged huNSG-QUAD mice. **(A, B)** At 6 weeks post-engraftment, sex-matched huNSG-QUAD littermates were injected intraperitoneally with vehicle or with 50 mg/kg MCC950 and injected intraperitoneally 1 hour later with PBS or with 15 μg LPS. Indicated cytokines were measured in **(A)** spleen and **(B)** liver at 6 hours after the challenge. All data represent means ± SEM with dots representing individual mice, and different colors representing different HPSC donors. The dotted line on each graph represents the murine background level of the respective ‘human protein’ detected in respective samples from an LPS-injected non-humanized NSG-QUAD mouse using this assay. Statistics were analyzed by a two-way ANOVA on log-transformed data followed by Sidak’s multiple comparison tests. *** p<0.001; ns not significant.

Supplementary Figure 6 Flow cytometric analysis of human monocyte viability in peripheral blood of HSPC-reconstituted NSG-QUAD mice. Representative flow-cytometric plots to assess viability of human monocytes (pre-gated as in **Supplementary Figure 1A**) in peripheral blood of huNSG-QUAD mice at week 6 post-engraftment, challenged as in **Figure 4** with PBS, LPS or LPS + MCC950. FSC, forward scatter.
