# Supplementary figures and images for: Development of human innate immune responses in a humanized mouse model expressing four human myelopoiesis transgenes
Source: Front Immunol. 2024 Sep 27;15:1419117. doi: 10.3389/fimmu.2024.1419117 (PMC11466769; doi:10.3389/fimmu.2024.1419117)

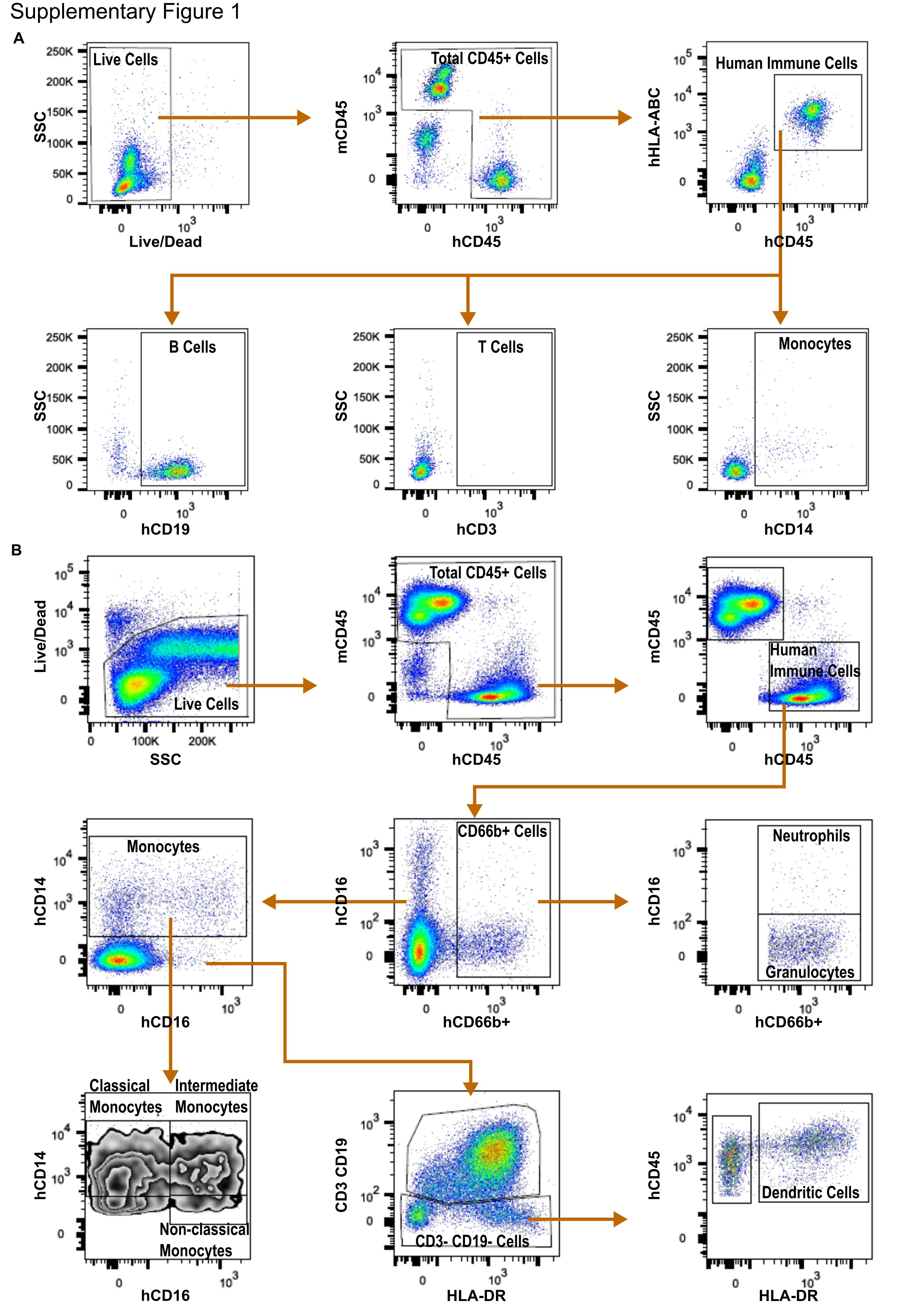

Supplement: Supplementary Figure 1 — Flow cytometric analysis of human immune cells in HSPC-reconstituted NSG-QUAD mice. (A) Representative flow-cytometric gating to assess human immune cell reconstitution in peripheral blood at week 5 post-engraftment (as in Figure 1B ). Doublets and dead cells were excluded before identifying live cells. From total mCD45+ and hCD45+ cells, human immune cells were identified as hCD45+hHLA+ cells. From this latter population, human B cells were identified as hCD19+ cells, human T cells were identified as hCD3+ cells, and human monocytes were identified as hCD14+ cells. SSC, side scatter. (B) Representative flow-cytometric gating to assess human myeloid immune cell reconstitution in peripheral blood at week 6 post-engraftment (as in Figures 1C–H ). Doublets and dead cells were excluded before identifying live cells. From total mCD45+ and hCD45+ cells, human immune cells were identified as hCD45+ cells. From this latter population, the hCD66+ fraction was further identified as hCD16+ neutrophils and hCD16- granulocytes; while the hCD66- fraction was further identified as hCD14+ monocytes that were classified into different subtypes based on hCD14 and hCD16 expression levels. From the remaining hCD45+hCD66-hCD14- fraction, hCD19+ B and hCD3+ T cells were excluded, after which hHLA-DR+ cells were identified as dendritic cells. SSC, side scatter. [file Image1.jpeg]

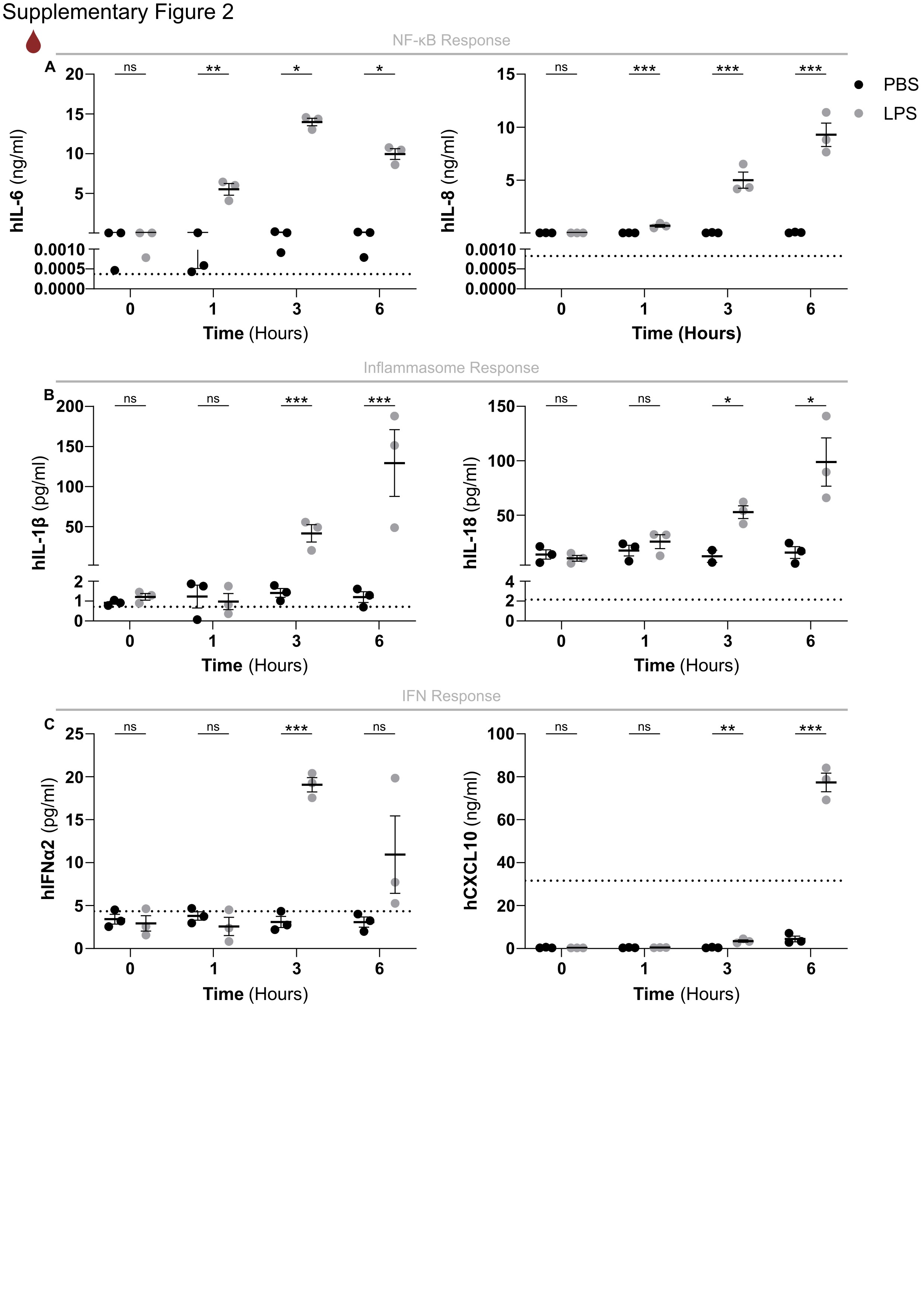

Supplement: Supplementary Figure 2 — Kinetics of human NF-κB- and inflammasome-dependent cytokine responses as well as human type I IFN responses in huNSG-QUAD mice upon a systemic LPS challenge. (A–C) Sex- and age-matched huNSG-QUAD littermates were injected intraperitoneally with 15 μg LPS at 6 weeks post-engraftment. Blood was collected before the challenge as well as after 1, 3 and 6 hours. Indicated human (A) NF-κB-dependent cytokines, (B) inflammasome-dependent cytokines, and (C) type I IFN response indicators were measured in serum. All data represent means ± SEM with dots representing individual mice reconstituted with HSPCs from the same donor. The dotted line on each graph represents the murine background level of the respective ‘human protein’ detected in serum samples from an LPS-injected non-humanized NSG-QUAD mouse using this assay. Statistics were analyzed by an unpaired t test or Mann Whitney test on log-transformed data. * p<0.05; ** p<0.01; *** p<0.001; ns not significant. [file Image2.jpeg]

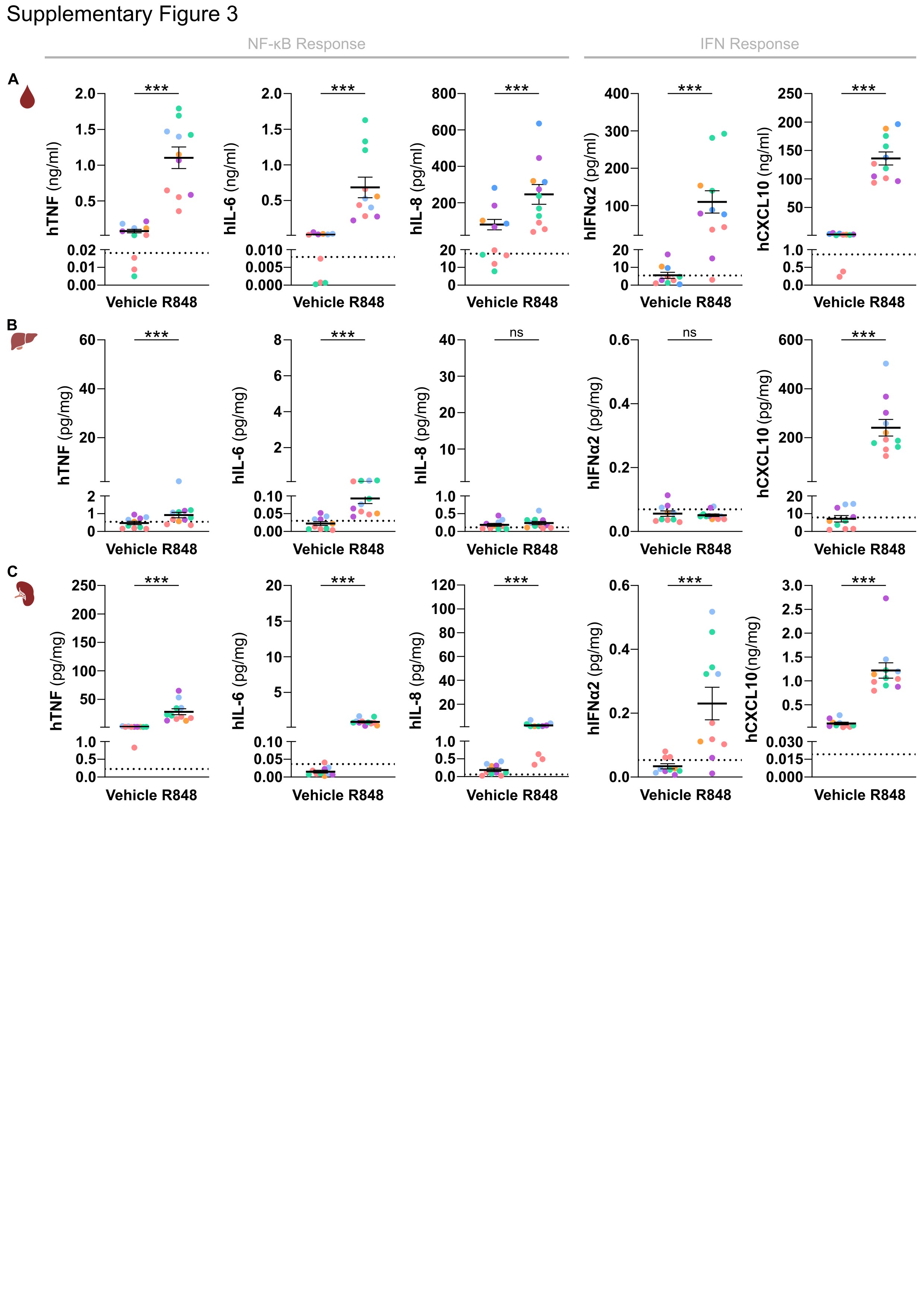

Supplement: Supplementary Figure 3 — HuNSG-QUAD mice display human NF-κB cytokine and type I IFN responses upon a systemic R848 challenge. (A–C) Sex- and age-matched huNSG-QUAD littermates were injected intraperitoneally with vehicle or with 200 μg R848. Indicated human proteins were measured in (A) serum, (B) liver, and (C) spleen 6 hours after the challenge. All data represent means ± SEM with dots representing individual mice, and different colors representing different HPSC donors. The dotted line on each graph represents the murine background level of the respective ‘human protein’ detected in respective samples from an R848-injected non-humanized NSG-QUAD mouse using this assay. Statistics were analyzed by a two-way ANOVA on log-transformed data followed by Sidak’s multiple comparison tests. *** p<0.001; ns not significant. [file Image3.jpeg]

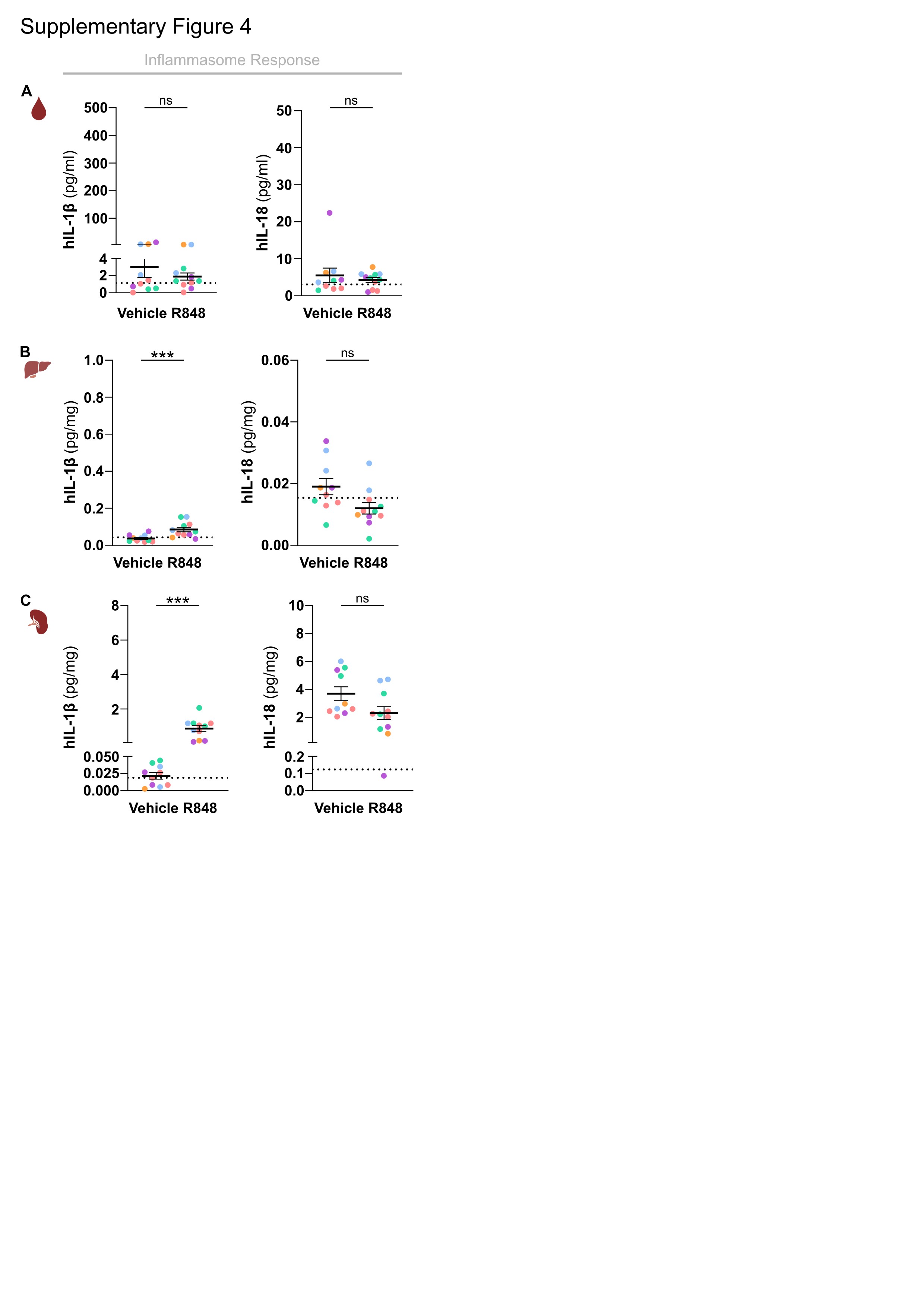

Supplement: Supplementary Figure 4 — HuNSG-QUAD mice display limited human inflammasome responses upon a systemic R848 challenge. (A–C) Sex- and age-matched hu-NSG-QUAD littermates were injected intraperitoneally with vehicle or with 200 μg R848. Inflammasome-generated hIL-1β and hIL-18 were measured in (A) serum, (B) liver, and (C) spleen 6 hours after the challenge. All data represent means ± SEM with dots representing individual mice, and different colors representing different HPSC donors. The dotted line on each graph represents the murine background level of the respective ‘human protein’ detected in respective samples from an R848-injected non-humanized NSG-QUAD mouse using this assay. Statistics were analyzed by a two-way ANOVA on log-transformed data followed by Sidak’s multiple comparison tests. *** p<0.001; ns not significant. [file Image4.jpeg]

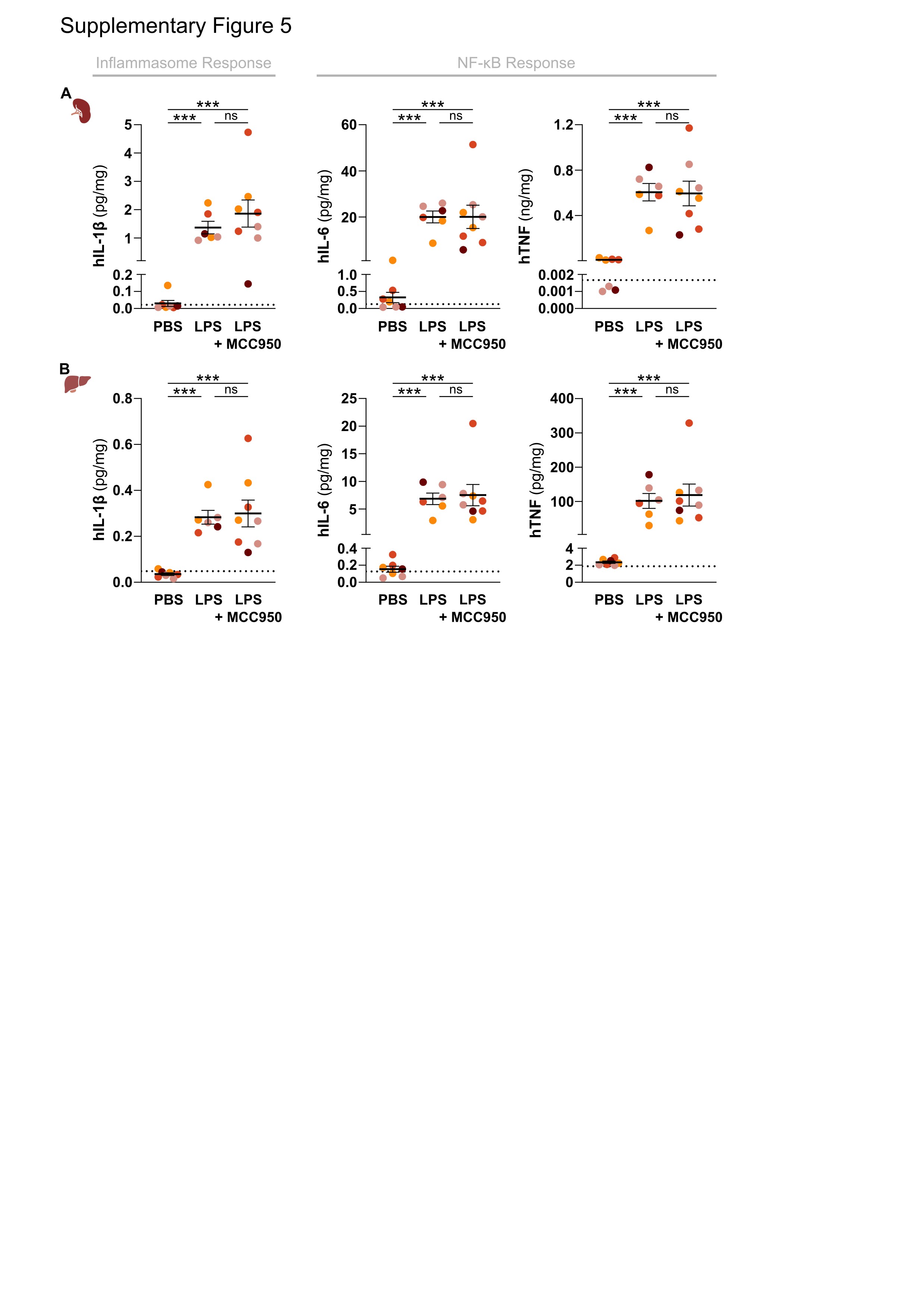

Supplement: Supplementary Figure 5 — Splenic and hepatic hIL-1β production is not affected by MCC950 treatment in LPS-challenged huNSG-QUAD mice. (A, B) At 6 weeks post-engraftment, sex-matched huNSG-QUAD littermates were injected intraperitoneally with vehicle or with 50 mg/kg MCC950 and injected intraperitoneally 1 hour later with PBS or with 15 μg LPS. Indicated cytokines were measured in (A) spleen and (B) liver at 6 hours after the challenge. All data represent means ± SEM with dots representing individual mice, and different colors representing different HPSC donors. The dotted line on each graph represents the murine background level of the respective ‘human protein’ detected in respective samples from an LPS-injected non-humanized NSG-QUAD mouse using this assay. Statistics were analyzed by a two-way ANOVA on log-transformed data followed by Sidak’s multiple comparison tests. *** p<0.001; ns not significant. [file Image5.jpeg]

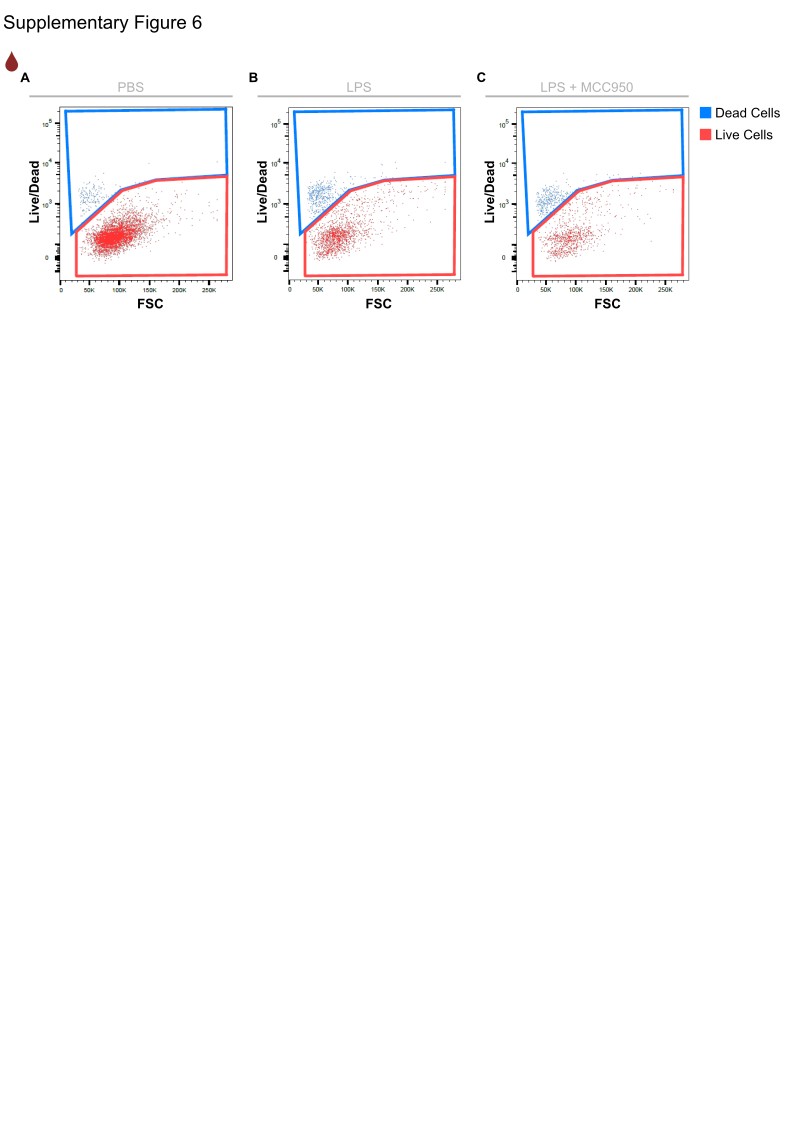

Supplement: Supplementary Figure 6 — Flow cytometric analysis of human monocyte viability in peripheral blood of HSPC-reconstituted NSG-QUAD mice. Representative flow-cytometric plots to assess viability of human monocytes (pre-gated as in Supplementary Figure 1A ) in peripheral blood of huNSG-QUAD mice at week 6 post-engraftment, challenged as in Figure 4 with PBS, LPS or LPS + MCC950. FSC, forward scatter. [file Image6.jpeg]
